# Splenic Injury Following Endoscopic Retrograde Cholangiopancreatography – A Role for Angioembolization

**DOI:** 10.5334/jbsr.2843

**Published:** 2022-08-11

**Authors:** Divyansh Agarwal, Philicia Moonsamy, Carlos Fernandez-Del Castillo

**Affiliations:** 1 Massachusetts General Hospital, US; 2 Harvard Medical School, US

**Keywords:** ERCP, Splenic hematoma, angioembolization, jaundice

## Abstract

**Teaching Point:** Splenic injury is an uncommon complication after endoscopic retrograde cholangiopancreatography (ERCP), requiring a high degree of suspicion in a patient who develops abdominal pain and/or hypotension after ERCP; in the appropriately selected patient splenic angioembolization can be the first-line treatment option.

## Case History

**Figure 1:**
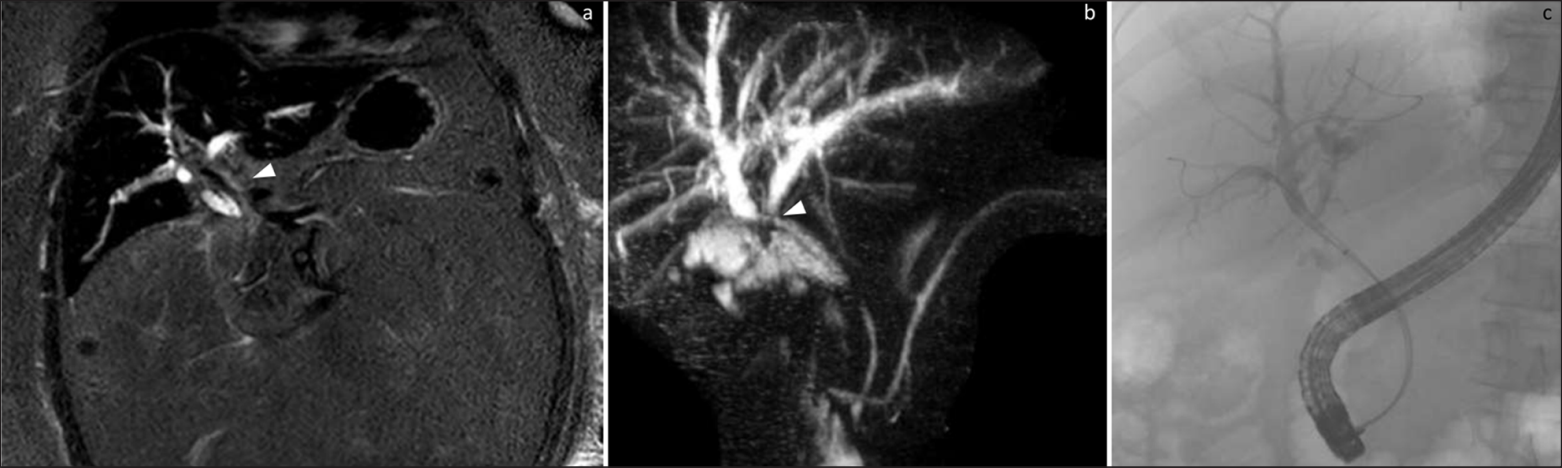


A 63-year-old man presented to the emergency department with five days of constant right upper quadrant pain, nausea, fevers, chills, fatigue, and jaundice. His physical exam was notable for scleral icterus, and a positive Murphy’s sign, and his labs at presentation were notable for a white blood cell count of 13.3, a total bilirubin level of 5.7, and alkaline phosphatase level of 435. Magnetic resonance cholangiopancreatography (MRCP) revealed a mass at the porta hepatis near the common bile duct origin, with moderate upstream intrahepatic biliary ductal dilatation (Figure 1 a–b, arrowhead). This was further evaluated the following day via an endoscopic retrograde cholangiopancreatography (ERCP) (Figure 1c).

**Figure 2:**
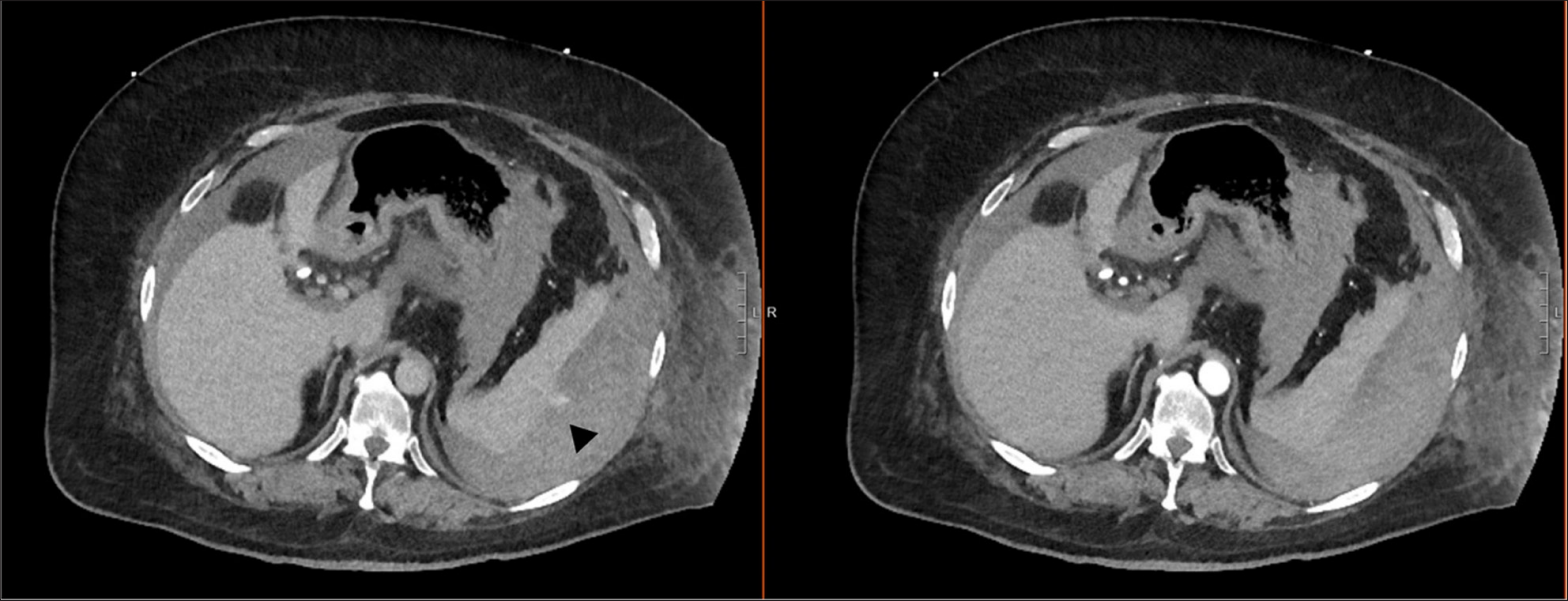


During the ERCP, biliary sphincterotomy and hilar stricturoplasty were performed, the right main hepatic duct was dilated with a 4mm balloon, and a plastic stent was placed. Four days after the procedure, the patient developed severe left upper quadrant abdominal pain, hypotension, altered mental status, and anemia. An urgent contrast-enhanced computed tomography (CT) of the abdomen (Figure 2) revealed a subcapsular splenic hematoma with active extravasation of contrast (black arrow), and moderate-sized hemoperitoneum predominantly along the spleen and extending into the left paracolic gutter and pelvis.

**Figure 3:**
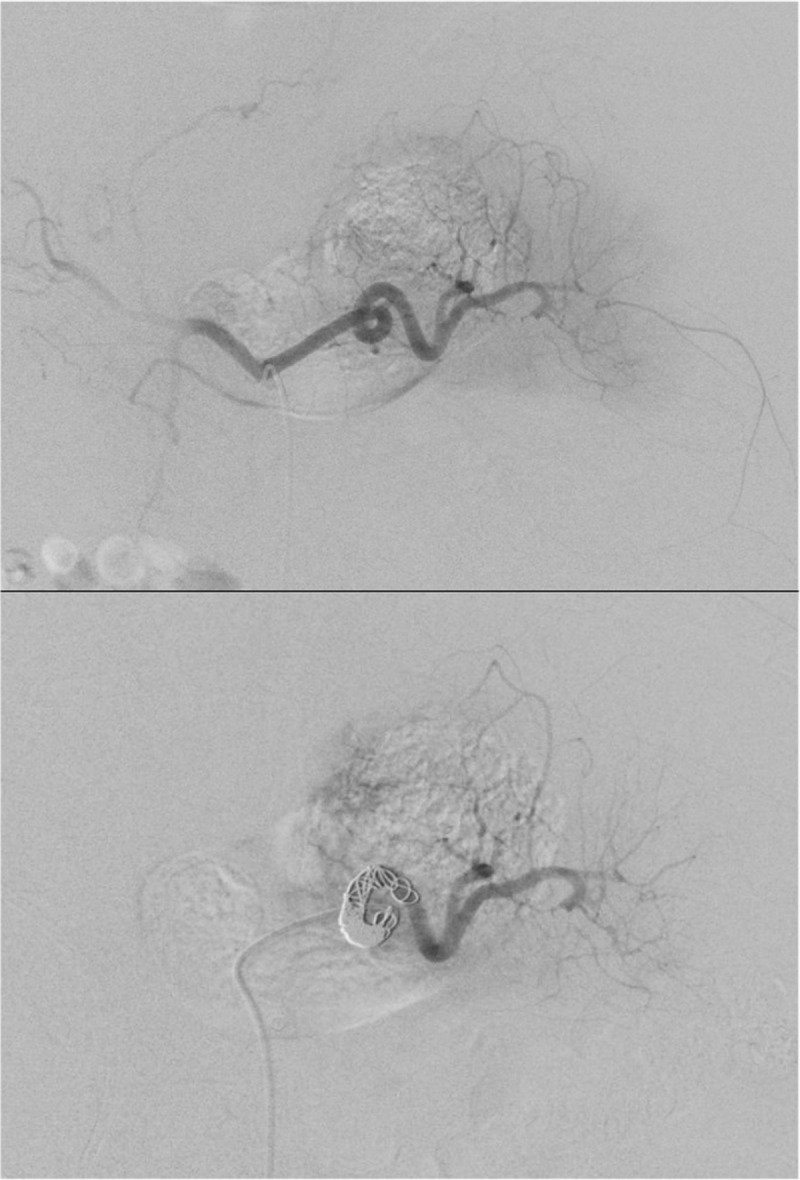


The decision was made to address the patient’s splenic injury via a minimally invasive approach over a surgical splenectomy. Consequently, the patient was successfully treated with proximal splenic embolization using coils, resulting in reduced splenic artery flow (Figure 3). Angiography confirmed minimal filling of the splenic artery and extensive reflux, suggestive of successful embolization.

The procedure was well tolerated, without any complications. Three weeks later, repeat CT imaging showed a stable peri-splenic hematoma with multiple new splenic infarcts post embolization.

## Commentary

ERCP remains an important diagnostic and therapeutic intervention for a wide array of hepatobiliary and pancreatic conditions. While complications such as pancreatitis are commonly known to be associated with ERCP, much less common is the possibility of splenic injury. Splenic hematoma or hemorrhage can result from excessive endoscope-related direct, traction or shear forces [1]. A paucity of awareness of this rare complication can often result in a delay in its diagnosis, therefore clinicians should have a high degree of suspicion for splenic injury as a diagnosis in patient with hemodynamic instability after an endoscopy.

Angioembolization can be a viable first-line intervention for managing splenic hematoma or hemorrhage after ERCP, prior to considering more invasive surgical operations such as splenectomy.
